# Expression analysis of *ABCA1* in type 2 diabetic Pakistani patients with and without dyslipidemia and correlation with glycemic index and lipid profile

**DOI:** 10.1038/s41598-023-43460-9

**Published:** 2023-10-11

**Authors:** Amber Zaidi, Amir Rashid, Asifa Majeed, Ayesha Naeem, Wajeeha Akram, Zunaira Ali Baig

**Affiliations:** Department of Biochemistry and Molecular Biology, Army Medical College, National University of Medical Sciences (NUMS), Rawalpindi, Pakistan

**Keywords:** Biochemistry, Genetics, Molecular biology, Endocrinology

## Abstract

Diabetes Mellitus type II, earlier considered as an endocrinological disorder is now more regarded as an inflammatory disorder along with lipid aberrations. It demands for regular monitoring, healthy dietary habits and lifestyle modification. This study was focused on gene expression of ATP binding cassette protein 1 (ABCA1) in diabetic dyslipidemia patients in comparison with control groups of only diabetics and healthy individuals. Blood samples and data were collected from recruited 390 patients who were further divided into three groups (130 each). Glycemic index and lipid profile was assessed. Delta Delta Ct method was used that revealed downregulation of the studied gene more in diabetic dyslipidemia patients as compared to only diabetics and healthy controls. The Ct values of *ABCA1* were associated with glycemic index and lipid profile using Pearson’s correlation. A negative correlation with fasting blood sugar and a positive correlation with HbA1cwas observed in only diabetics group. While in diabetic dyslipidemia and normal healthy controls, a negative correlation was found with both. As far as the lipid profile is concerned a positive correlation was observed among only diabetics with whole lipid profile. In diabetics with dyslipidemia, a negative correlation with all parameters except the TAGs was observed. A positive correlation with all except HDL was observed in healthy controls. The Ct values and fold change were compared among diseased and healthy individuals by applying independent t test. The cycle threshold in only diabetics was *p* = 0.000018 and in diabetic dyslipdemia individuals was *p* = 0.00251 while fold change in only diabetics (*p* = 0.000230) and in diabetics with dyslipidemia (*p* = 0.001137) was observed to be as statistically significant.

## Introduction

Diabetes Mellitus (DM) is a hyperglycemic metabolic disorder characterized by insulin deficiency, insulin resistance or both. It is further categorized into various types^1^. The global prevalence of diabetes is expected to rise to 629 million by year 2045^2^. According to International Diabetes Federation (IDF) Atlas 2022, 10th edition about 90 million people in South East Asia are suffering from type 2 diabetes mellitus^3^. Basit et al. reported 26.3% prevalence of diabetes in Pakistan among which 7.1% were newly diagnosed cases which marks it a national epidemic^4^. Dyslipidemia that was considered as a predictor of cardiovascular disease is now under study as a risk factor for diabetes mellitus type 2 as well^5^. In South Asia and South East Asia, the prevalence of obesity is expected to be doubled by the year 2030^6^. High circulating levels of cholesterol and triacylglycerides (TAGs) and low levels of high-density lipoprotein (HDL) altogether constitute for dyslipidemia^7^. About 70% of diabetic patients have been reported to be affected from dyslipidemia^8^. Among poorly controlled diabetic patients, dyslipidemia has been reported to be markedly prevalent^9^. Glycosylated hemoglobin (HbA1c) a well-known diagnostic marker of diabetes mellitus, has also been declared as an early predictor of dyslipidemia^10^. ABCA1 being widely expressed in liver, GIT, adipose tissue and macrophages; is considered a crucial therapeutic target to treat diabetic dyslipidemia^11^.

The ABCA1 stimulators can decrease lipid accumulation in blood preventing dyslipidemia^12^. Lipid profiles and multiple lipid ratios have been reported to be predictors of glycemic control among type II DM patients^13^. Papers on glycemic status and lipid profile have been published but no such research related to the expression of *ABCA1* has yet been reported on Pakistani population. The aim of this research was to investigate the expression analysis of *ABCA1* gene in Pakistani population and to find its correlation with glycemic status and lipid profile of diabetics, diabetics with dyslipidemia and healthy controls.

## Results

### Biochemical and molecular analysis

The recruited study participants were assessed for glycemic index, fasting blood sugar (FBS) and HbA1c while in lipid profile total cholesterol (TC), TAGs, low-density lipoproteins (LDL) and HDL. The *ABCA1* gene RNA expression was measured through RT-PCR. The downregulation of *ABCA1* expression was observed more in diabetics with dyslipidemia group as compared to only diabetics and healthy control groups. Delta Delta Ct method by Livak was used to assess the cycle threshold values^14^.

### Correlation of gene expression with glycemic index and lipid profile

**Figure 1:**
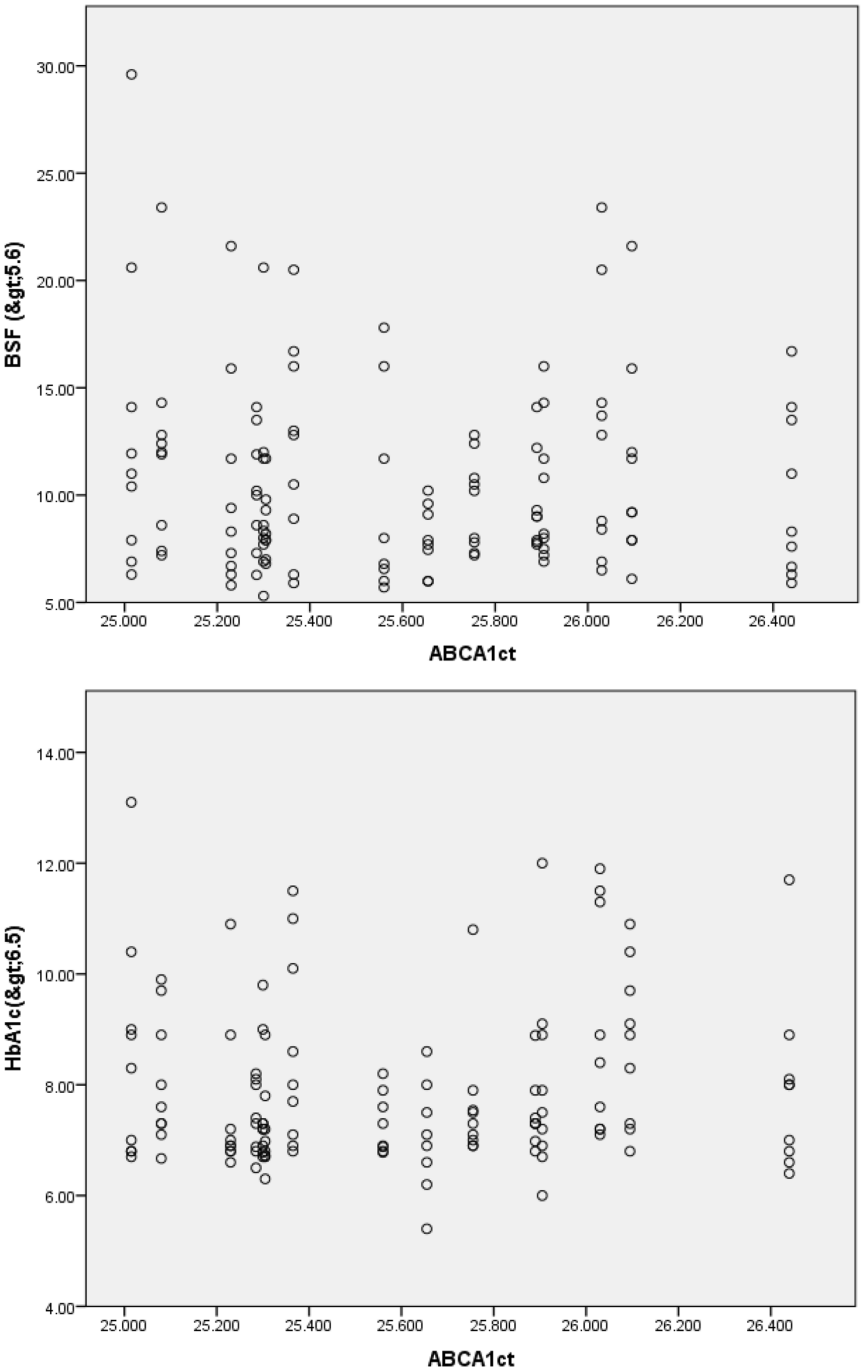
Pearson’s correlation of *ABCA1* Ct of only diabetics with l fasting blood sugar and HbA1c.

**Figure 2:**
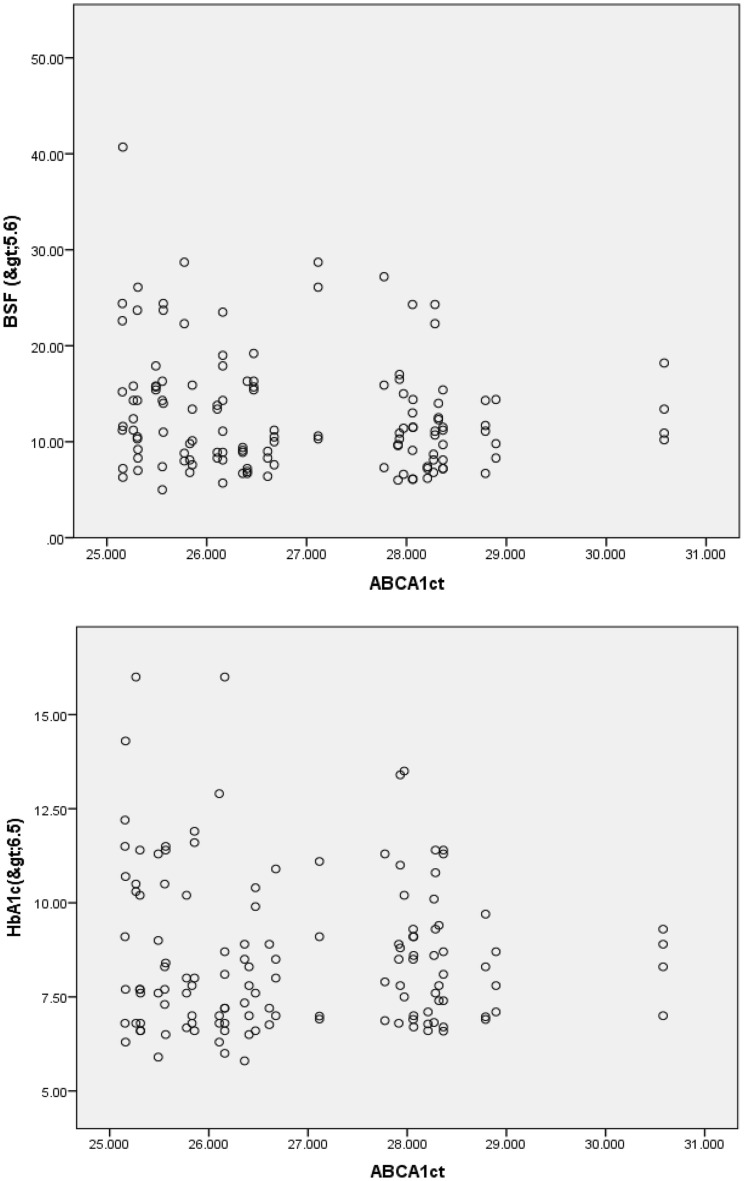
Pearson’s correlation of ABCA1 ct of diabetic dyslipidemia with fasting blood sugar and HbA1c.

Pearson’s correlation was applied to analyze the correlation of cycle threshold value of *ABCA1* with glycemic index and lipid profile (Table 1). Two tailed significance was shown as positive in only diabetics group (Fig. 1).A negative correlation of *ABCA1* expression was found with fasting blood sugar while a positive correlation was found with HbA1c. In context of lipid profile, a positive correlation was seen with all the lipid parameters. In the diabetic dyslipidemia group, a negative correlation of expression was seen with both fasting blood sugar and glycosylated hemoglobin. For the lipid profile, a positive correlation was found with TAGs while a negative correlation was seen with rest of the parameters i.e. TC, HDL and LDL. In healthy controls, a negative correlation of *ABCA1* expression was seen with both FBS and HbA1c, while a positive correlation was seen with lipid parameters i.e. TAGs, TC and LDL except HDL showing a negative correlation (Fig. 2).

**Table 1:** Pearson’s correlation of *ABCA1* Ct value of the study groups with lipid Profile.

*ABCA1* (Ct)	Total cholesterol	TAGs	LDL	HDL
Only diabetics	0.059	0.022	0.030	0.012
Diabetic Dyslipidemia	− 0.268	0.022	− 0.213	− 0.064
Healthy control	0.022	0.041	0.081	− 0.098

### Association of *ABCA1* Ct values of cases and controls by applying independent t test

Independent t test was applied to compare means of *ABCA1* Ct values (Table 2) and fold change (Table 3) of only diabetics and diabetic dyslipidemia patients with normal healthy controls. The n = 10 and two tailed significant values were considered.

**Table 2:** Comparing means of only diabetics and diabetic with dyslipidemia with healthy controls in context of *ABCA1* Ct by applying independent t test.

Groups	Only diabetics (mean ± SD)	Healthy controls (Mean ± SD)	*p* Value
*ABCA1*Ct	25.62 ± 0.488	24.369 ± 0.481	0.000018

	Diabetic dyslipidemia (mean ± SD)	Healthy controls (Mean ± SD)	
*ABCA1* Ct	27.19 ± 1.57	24.369 ± 0.481	0.002511

**Table 3:** Comparing means of only diabetics and diabetics with dyslipidemia with healthy controls in context of fold change by applying independent t test.

Groups	Only diabetics (means ± SD)	Healthy controls (Mean ± SD)	*p* Value
Fold change	3.05 ± 1.27	11.84 ± 6.75	0.000230

	Diabetic dyslipidemia (means ± SD)	Healthy controls (Mean ± SD)	
Fold change	2.045 ± 2.67	11.84 ± 6.75	0.001137

**Figure 3:**
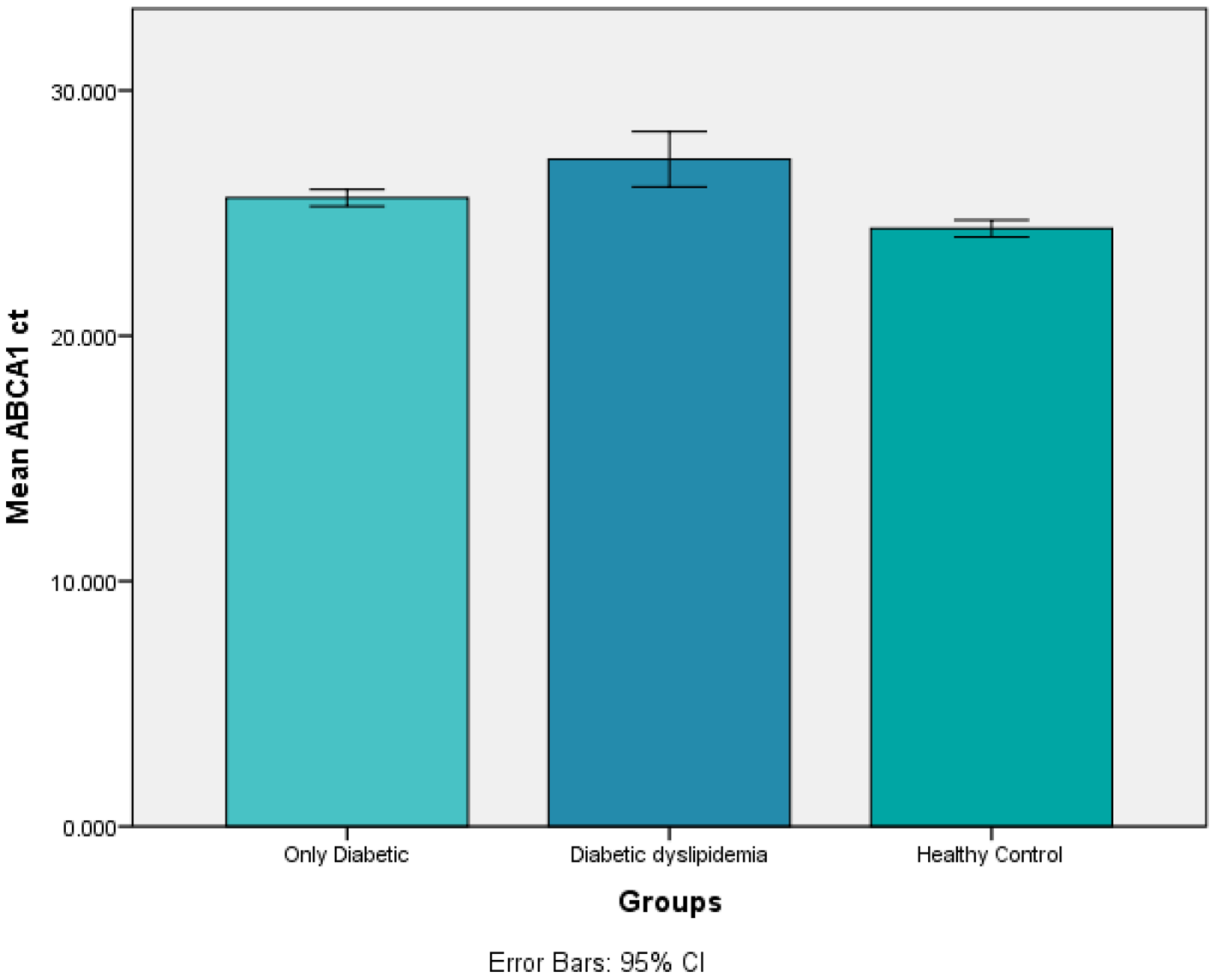
Histogram showing the results of independent t test for ABCA1 Ct among the three groups.

**Figure 4:**
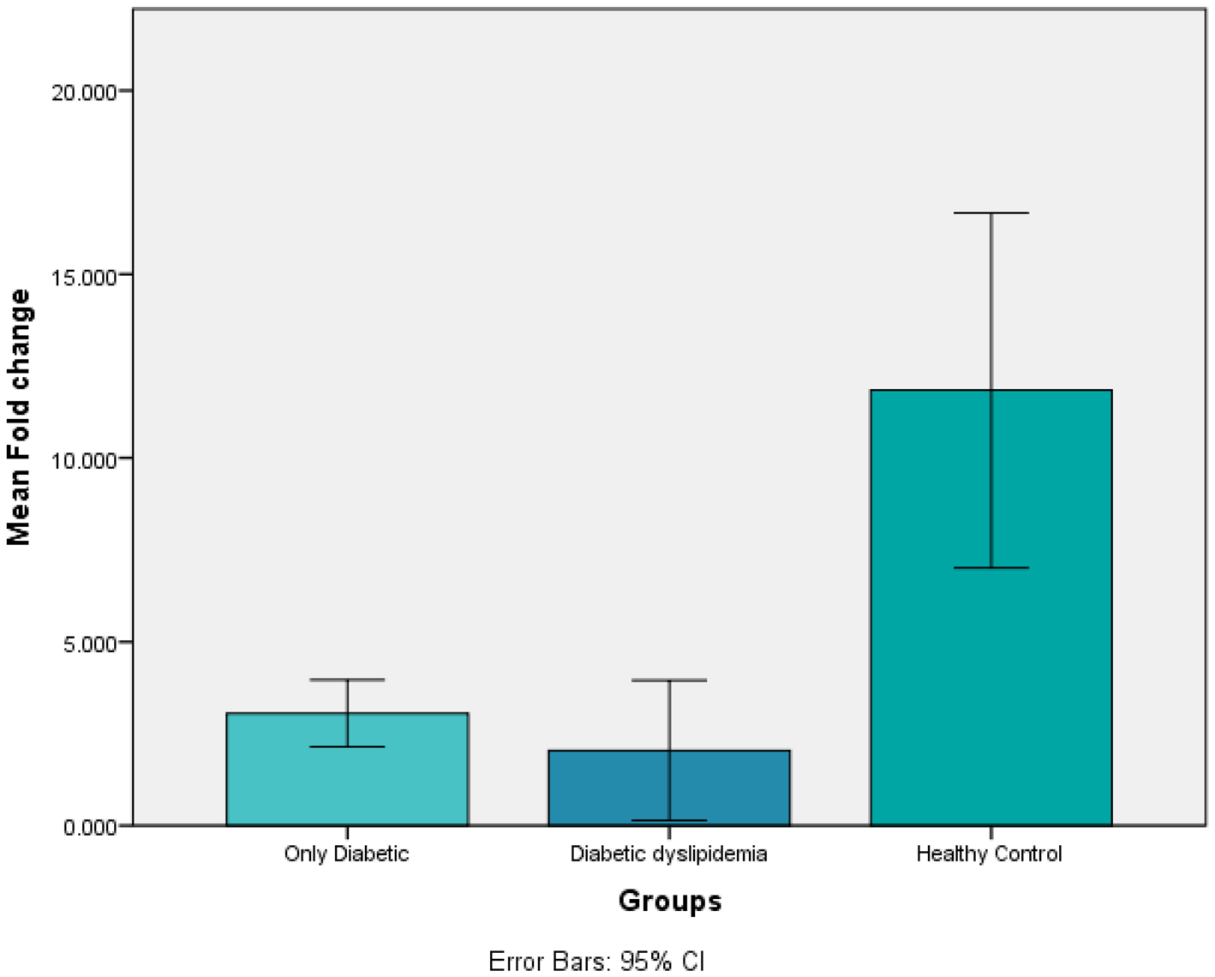
Histogram showing the results of independent t test for fold change among the three groups.

Both the *ABCA1* Ct values (Fig. 3) and fold change (Fig. 4) were found to be statistically significant (*p* =  < 0.05) among the two groups of only diabetics and diabetics with dyslipidemia by comparing the means via independent t test.

## Discussion

In our study, the downregulation of *ABCA1* gene was observed among patients of diabetes and diabetic dyslipidemia as compared to normal healthy individuals. We demonstrated a negative correlation of glycemic index in all three groups except only diabetics. In context of lipid profile, only diabetics displayed positive correlation of *ABCA1* with most of its parameters while the rest of two groups mostly gave negative correlations. These results are in accordance with multiple studies already conducted and published worldwide. Our findings suggest the association of low *ABCA1* with that of lipid profile and hyperglycemia. In line with our findings, low expression of the gene in adipose tissue has been reported to be associated with insulin resistance and obesity^15^. Several variations in the gene have also been observed to have to role in HDL level alteration constituting varied blood sugar levels, and it has also been highlighted in a study that managing dyslipidemia can prevent further progression of diabetes Mellitus^16–18^.*ABCA1* has also been presented as a repressor of inflammation which is a contributor in the baseline pathology of DM^19^. *ABCA1* genetic mapping has been proposed to manage lipid levels among type II DM patients^20^. On the other hand, some variations in *ABCA1* have been found to have beneficial lipid effects on both males and females^21^.

In our study, we have observed a negative correlation of gene expression with the fasting blood sugar among diabetic subjects, while among diabetic dyslipidemia subjects, both the fasting blood sugar and lipid parameters showed a negative correlation except for TAGs. These findings are in accordance with a study which also revealed reduced *ABCA1* expression in type 2 diabetic study participants^22^. HbA1c has been found to be a good diagnostic tool in cases of prediabetes and diabetes in the country, as well as a good predictor of lipid profile^23–25^. A positive association of HbA1c has been reported with macro and microvascular complications of diabetes mellitus type 2^26^. In type II diabetics triglyceride–glucose index has been reported to be significantly associated with HbA1c levels and thus is a potential predictor of glycemic control^27^. Moreover HbA1c a universal diagnostic marker of DM has also been reported as a significant indicator of dyslipidemia in diabetic patients^28^. Our findings exhibited diminished levels of *ABCA1* correlated with that of lipid profile and fasting blood sugar among subjects with dyslipidemia. Similarly, a study reported a strong positive association of deranged glycemic status with dyslipidemia among diabetic patients^29^. On the contrary Maria et al. reported in her study that there is no significant relationship of HbA1c with lipid profile among Pakistani patients with type 2 diabetes mellitus^30^.

With reference to lipid profile of type II diabetic patients, LDL levels have been manifested to be high and HDL levels as low^31^. Obesity and overweight have been positively associated with dyslipidemia and diabetes^32^. Our study findings couldn’t establish a positive correlation of TAGs with that of gene expression in deranged lipid and diabetic subjects while a significant difference was observed in levels of triglycerides with that of diabetics in a study^33^. The prevalence of dyslipidemia has been observed to be increased in 60–70% patients of type II diabetes mellitus, being low HDL level dominant in worsening the glucose tolerance among diabetics^34,35^. A study conducted on Pakistani population reported that lipid parameters are alarmingly high in type 2 diabetes mellitus specially among the newly diagnosed ones^36^. Akhtar et al. reported that type 2 DM patients had marked dyslipidemia with TGs and HDL levels being most deranged among all^37^. A Taiwan based study reported association of high levels of HbA1c with dyslipidemia in diabetic patients^38^. However, TG to HDL ratio has been positively associated with type 2 DM^39^. Baig et al. reported that dyslipidemia is an early transition in type 2 DM rather than a later stage complication^40^. In another study increased TGs and LDL levels have been reported to be associated with high levels of HbA1c in young type 2 diabetics^41^.

Our study results are demonstration of relationship between *ABCA1* gene RNA expression and HbA1c, fasting blood sugar both in diabetics and non-diabetics. The findings suggest that diabetes mellitus is associated with dyslipidemia through diminished *ABCA1* gene expression. The findings from our study will contribute in genetic pool of studies concerning *ABCA1* in diabetes and diabetic dyslipidemia. Therefore, it will be helpful in designing new prevention techniques and life style modifications to slow down the progression of type II diabetes mellitus and its complications. As a result, our future generation, genetically at extreme risk of the disease, can be salvaged from this lifetime debilitating illnesses. The limitations of our study were a short budget; expression analysis could have been performed on a larger sample size and among various ethnic groups to assess the effect of diet and life styles on it. In future the signaling pathways of *ABCA1* can be targeted with special reference to their pharmacogenomic and pharmacokinetic aspects. This can be a revolutionary step in field of personalized medicine to uproot type II Diabetes Mellitus from our future generations.

## Conclusion

*ABCA1* gene expression was more downregulated in diabetics with dyslipidemia as compared to only diabetics and healthy controls. With glycemic index it is positively correlated among only diabetics and negatively correlated in both diabetics with dyslipidemia and healthy controls. With lipid profile it is positively correlated in both only diabetics and healthy controls and negatively correlated among diabetics with dyslipidemia. Hence downregulation of the gene is directly related to both glycemic index and lipid profile and ABCA1 transporter protein deficiency markedly affects levels of both of these parameters in the plasma. The independent t test results were statistically significant (*p* =  ≤ 0.05) when observed in relation with *ABCA1* Ct and fold change among only diabetics and diabetics with dyslipidemia as compared to normal healthy.

## Materials and methods

A formal approval of this research study was obtained from ethical review committee of Army Medical College, ref number **(**ERC/ID 04, dated: 2 Jan, 2019**).** All methods were performed in accordance with the relevant guidelines and regulations. The approval for patient enrollment, data collection and blood sample collection was taken from Pathology Department of Pak Emirates Military Hospital (PEMH), Rawalpindi. Informed consent in written was taken from all patients enrolled. A systematic questionnaire was designed and data was collected from all recruited study subjects (n = 390) using non probability purposive sampling. This is a cross sectional comparative study. Clinically diagnosed patients of type II Diabetes Mellitus and Diabetic dyslipidemia were enrolled with consultation of Medical Specialist at PEMH. Confidentiality codes were assigned to all enrolled patients and their samples and reports were later followed according to them. The study subjects were further categorized into three groups (n = 130 in each group)as diabetics with dyslipidemia (group1), only diabetics (group II) and 130 as healthy controls (group III). The inclusion criteria for patients was freshly diagnosed patients of diabetes mellitus type 2 and diabetic dyslipidemia of ages > 30 years. Both genders were included in the study. Patients with only dyslipidemia, having any chronic illness like hypertension and cardiovascular illnesses and those on any anti-diabetic medication and lipid lowering therapy were particularly excluded from the study. Age and gender matched normal healthy controls were enrolled in the study.

### Blood sampling and parameters analysis

A total of 5 ml of venous blood samples were drawn from patients enrolled in all three groups. The biochemical parameters assessed were fasting blood sugar, HbA1c and complete lipid profile including total cholesterol, triglycerides, low density lipoprotein and high density lipoprotein.

### RNA extraction and cDNA synthesis

RNA was extracted using TRIzol reagent by Invitrogen within 4–6 h of sampling and extracted RNA samples were saved at − 80 °C. The primers of cDNA were designed on Primer 3 plus (available at https://www.primer3plus.com/index.html) according to the previously available sequences of *ABCA1* available from National Centre for Biotechnology information (NCBI).

The sequences of the primers used are mentioned hereby.

### Forward primer

CTGGCCAGGATATTCAGCAT

### Reverse primer

TGAGAACTGCAACGTCCACT

For comparison in expression analysis, *GAPDH* was considered as a house keeping gene among all enrolled patients. The program was optimized on 96 well T100 Thermal Cycler (BioRAD). Respective cDNA from all extracted RNA samples were prepared using RevertAid First stand cDNA synthesis kit (Thermoscientific USA). Samples were further processed for expression analysis on CFX Touch 96 well Real-time PCR detection system (BioRAD). Maxima SYBER Green Master mix with ROX was used (Thermoscientific USA). For relative quantification ∆∆Ct method was used.

### Statistical analysis

Data was analyzed using SPSS version 22. For gene expression analysis ∆∆Ct method by Livak^14^ was used. For correlation of the expression with glycemic index and lipid profile Pearson’s correlation test was applied. For comparison of Ct values and fold change of *ABCA1* among the three groups, independent t test was applied.

## Data Availability

Data is available from corresponding author on reasonable request.
